# Flow cytometry immunophenotyping of peripheral blood circulating B-cell subset: comparison of osteoarthritis, chronic lymphocytic leukemia patients and normal donors

**DOI:** 10.1186/1479-5876-9-S2-P20

**Published:** 2011-11-23

**Authors:** Artjoms Spaks, Irina Spaka, Alla Rivkina

**Affiliations:** 1 A. Kirchenstein Institute of Microbiology and Virology, Riga Stradins University, Riga, Latvia; 2 Hematology Dept. Riga Eastern University Hospital, Riga, Latvia

## Introduction

Peripheral blood (PB) circulating B-cell subsets have been poorly defined until ≥6-color flow cytometry (FC) became available [1]. Traffic of the B-cell subsets between tissues through PB reflects the immune status of an individual subject and potentially also disorders of B-cell development, autoimmunity, and lymphoproliferative diseases [2].

Chronic lymphocytic leukemia (CLL) is the most common type of leukemia in the Western world. It is a chronic lymphoproliferative disorder characterized by the accumulation of monoclonal B cells in the blood, bone marrow, and secondary lymphoid tissues [3]. Osteoarthritis (OA) is the most common articular disease worldwide. Immune system cells are involved in the matrix degradation that characterizes cartilage degeneration in OA [4].

## Aim

The aim of our study was to quantify and compare proportions of B-cell sub-populations in PB of CLL, OA patients and normal donors (ND).

## Patients and methods

PB samples were collected from 10 patients diagnosed with CLL, 4 patients diagnosed with OA and 9 ND. Conjugated monoclonal antibodies against CD19, CD10, CD27, CD38 and CD5 were used for immunophenotyping. Fluorescence measurements were made by BD FACS Aria II. BD FACS Diva 6.2 software was used to analyze data. Descriptive statistics were used to analyze data and summarize baseline characteristics.

## Results

Four subpopulations of B-cells were detected in peripheral blood samples: CD19+CD10+CD27-CD38+CD5++ immature B-cells - 20,7% (CLL), 41,2% (OA); 11,4% (ND); CD19+CD10-CD27+CD38+/-CD5- plasmatic B-cells – 5,4% (CLL), 9,4% (OA), 3,1% (ND); CD19+CD10-CD27++CD38++CD5- memory B-cells – 29,8 (CLL), 23,5% (OA), 28,4% (ND); CD19+CD10-CD27-CD38+/-CD5+/- naïve B-cells – 40,3% (CLL), 25.7% (OA), 56,8% (ND). Marked increase in immature and plasmatic B-cell number was found in OA and CLL patients.

## Conclusion

The frequency of immature and plasmatic B-lymphocytes in PB has been found to be increased in OA and CLL. Changes in B-cell subpopulation profile may be proposed as a disease development indicator.
